# Musculoskeletal Disorders in Portuguese Welders: Effects on Bodily Pain and Health-Related Quality of Life

**DOI:** 10.3389/fpubh.2021.660451

**Published:** 2021-05-20

**Authors:** Lilian Lourenço, Sílvia Luís

**Affiliations:** ^1^Universidade do Algarve, Faro, Portugal; ^2^Faculdade de Ciências Humanas e Sociais, Universidade do Algarve, Faro, Portugal; ^3^Centro de Administração e Políticas Públicas, Instituto Superior de Ciências Sociais e Políticas, Universidade de Lisboa, Lisboa, Portugal

**Keywords:** bodily pain, lumbar, quality of life, musculoskeletal disorders, welding

## Abstract

**Background:** Musculoskeletal disorders in welders may influence their health-related quality of life. However, few studies have addressed this issue and their results were inconclusive. This study investigates whether there are musculoskeletal disorders with a higher incidence in welders compared to non-welders, and whether these disorders lead to an increase in bodily pain which in turn decreases their health-related quality of life.

**Methods:** *A priori* analyses of statistical power were conducted to determine the sample size needed to find medium to large statistical effects, for a 0.05 alpha, and critical sampling, combined with snowball sampling, was carried out. The study was cross-sectional, and participants were asked to respond to a survey using validated instruments (*N*_*welders*_ = 40, *N*_*non*−*welders*_ = 42).

**Results:** As expected, a higher incidence of symptoms of musculoskeletal disorders in the cervical, dorsal, lumbar, and wrists and hands was found in welders in comparison to non-welders. Furthermore, the presence of musculoskeletal disorders, particularly in the lumbar area, was related to an increased bodily pain and decreased health-related quality of life.

**Conclusion:** Welders are exposed to a higher incidence of musculoskeletal disorders that decrease their quality of life. It is essential to increase the awareness of welders, organizations, and regulatory institutions toward this issue in order to motivate the development and implementation of prevention strategies. The need for primary and secondary prevention-type strategies, which have already proven their effectiveness in the context of welding, is highlighted.

## Introduction

Welders comprise a large occupational group that works for long hours in forced postures. The welding activities often imply that the workers remain statically, in the same posture, for long periods of time. It is often carried out by hand, requiring work in various positions, angles, and rotations to ensure high standards of product quality in accordance with the ISO standards. Maintaining an awkward posture can lead to work-related musculoskeletal disorders [e.g., (1–3)]. Musculoskeletal disorders represent a problem not only for the individual, but also for the organization and society. At a European level, the occurrence of musculoskeletal and connective tissue disorders represented over 17 million euros of losses in production (i.e., costs of lost production based on labor costs) in 2016 and more than 30 billion euros in the gross added value loss (i.e., loss of labor productivity) (4). To improve the quality of life of these individuals, as well as the sustainability of organizations and social security systems, it is essential to continuously assess and understand to what extent these professional activities and challenges influence the health-related quality of life of welders and to further invest in prevention strategies.

A substantial body of studies indicates that musculoskeletal disorders are associated with welding activities. For example, welders, in general, and professional divers working as welders have an increased risk of musculoskeletal complaints that are related to heavy physical demands at work (5). A study in the UK calculated the standardized incidence rate ratios for construction workers using work-related ill-health cases for individual job titles returned to The Health and Occupation Reporting network by clinical specialists and UK population denominators (6). A significant increase in the incidence of work-related ill-health cases was found, compared with other workers in the same major Standard Occupational Classification (i.e., workers with similar levels of qualifications, training, skills, and experience) for many conditions, including musculoskeletal disorders in welders.

Studies are not always consistent with the areas indicated as more prone to musculoskeletal disorders. For instance, a study conducted in Sweden comparing welders to office clerks and fishermen illustrated that welders have more musculoskeletal disorders overall, but particularly in the shoulder, using both self-reported data and objective signs of musculoskeletal disorders (2). In Iran, a study using self-reported data illustrated that 88% of the welders had musculoskeletal problems, predominantly on the knees, neck, and back (3). Another study in Iran illustrated that arc welders had significantly more musculoskeletal problems on the neck and wrists/hands regions than gas welders (7). In Shanghai, a study comparing welders to non-dust workers using self-reported measures indicated that welders have a more pronounced discomfort on their cervical vertebra and low back pain (8). These different results demonstrate the need for further research, as well as the need to consider the effects of anthropometric differences between countries.

The World Health Organization (WHO) defines health as a state of physical, mental, and social well-being, and not just the absence of disease or infirmity. Likewise, quality of life is the individual's perception of their position in life, according to their culture and value systems, taking into consideration their objectives, expectations, and concerns (9). The interest in the concept of quality of life in the health area is relatively recent and emerges from the new paradigms that have influenced practices and policies in the health sector in the last decades. Knowing an individual's own perception of health-related quality of life has become an important component of health surveillance, generally considered as a valid indicator for measuring care needs and monitoring the outcome of interventions [e.g., (8)]. Few studies have focused on the impacts of welding on the health-related quality of life. A cross-sectional study in the UK compared professional divers who had worked as a welder, professional welders who had not dived, and offshore oil field workers who had neither dived nor welded, but no differences were found in their physical quality of life. However, in the study conducted in Shanghai, authors found that the health-related quality of life was significantly worse in welders compared to non-dust workers (8). The authors suggest this difference occurred because the instrument they used to measure the health-related quality of life is commonly used and probably has a higher validity, namely the 36-item Short Form Health Survey, SF-36 (10). Of relevance for the present study, bodily pain was one of the dimensions that was significantly worse in welders (8).

This study had two main goals. The first was to analyze the musculoskeletal impacts of welding in Portuguese welders, comparing with a general population sample with similar sociodemographic characteristics. We expected to find a higher incidence of symptoms of musculoskeletal disorders among welders (Hypothesis 1). The second was to understand if the musculoskeletal disorders influenced the health-related quality of life due to bodily pain. To the best of our knowledge, this has not been analyzed in previous studies. We expected that musculoskeletal disorders with a higher incidence in welders decreased their health-related quality of life given that musculoskeletal disorders increase bodily pain (Hypothesis 2).

## Materials and Methods

### Sample Determination

*A priori* analyses of statistical power were carried out to determine the adequate sample size for a 0.05 alpha using the GPower software (11). To test Hypothesis 1, the total sample required for a chi-square test was 145 participants to find a medium effect and 52 participants to find a large effect (welders and not welders). To test Hypothesis 2, the total sample required for a linear regression model with two predictors was 107 welders to find a medium effect and 48 welders to find a large effect. Therefore, we defined that the minimum sample size would be 48 welders and 48 non-welders. A critical sampling process of welders was conducted, combined with snowball sampling, to recruit Portuguese welders and afterwards, to recruit a sample of non-welders with similar sociodemographic characteristics. Due to the ongoing pandemic, data collection was forced stop at 40 welders and 42 non-welders. To compensate, bootstrapping was used to test Hypothesis 2 given that this technique has a relatively more power to detect smaller statistical effects.

### Participants and Procedure

Forty welders and 42 non-welders employed by multiple Portuguese organizations read the informed consent and agreed to participate in the study. Critical and snowball sampling procedures were used to gather the sample: welding industries were initially identified then the welders were contacted by email and social media networks, and they were kindly asked to forward the study to other welders after responding to the survey. Most welders were male (95.0%), aged between 30 and 40 years old (40.0%), had a medium level education (97.5%), and technical training in welding (72.5%). They were mostly employed in (a) manufacturing industries (35.0%), (b) wholesale and retail trade; repair of motor vehicles and motorcycles (30.0%), and (c) electricity, gas, steam, hot and cold water, and cold air industries (22.5%).

The sample of non-welders was recruited online in various internet groups. We sought to recruit a sample of non-welders with similar sociodemographic characteristics. Therefore, the admission criteria for the non-welders were the following: an active worker, male, between 30 and 40 years old, and has a medium level of education. As a result, all non-welders were male and aged between 30 and 40 years old. However, only 83.3% had a medium level of education, with 16.7% indicating to have a higher-level education, most likely because they were completing their education at the time of the study. The study data was collected between February and October 2020.

### Measures

The survey for welders was composed of three sections: musculoskeletal disorders, bodily pain, and health-related quality of life. The survey for non-welders measured only the musculoskeletal disorders.

#### Musculoskeletal Disorders

A Portuguese version (12) of the Dutch Musculoskeletal Questionnaire (13) was used to identify which areas were most affected due to musculoskeletal disorders. To facilitate the identification of the anatomical zones, the questionnaire included a body diagram, highlighting the body regions, and participants were asked to mark which areas they felt discomfort, on a dichotomic scale of *yes* or *no*.

#### Bodily Pain

The bodily pain dimension of SF-36 (10, 14) was used to measure the intensity of the bodily pain and its effect on daily life (α = 0.69). It is composed of two items (e.g., “*During the last 4 weeks, how the pain has interfered with your normal work”)*, which participants responded on a scale that ranged from *1, absolutely nothing*, to *5, immensely*.

#### Health-Related Quality of Life

Health-related quality of life was measured using the SF-36 (10, 14). In this study, we used the following dimensions: physical function, social function, physical performance, and general health. To reduce the length of the questionnaire, we opted not use all the items of the survey as our aim was to use a composite measure of the health-related quality of life, not to study its dimensions. The physical function dimension (10 items) refers to the individual's ability to perform daily tasks, studying the impact of perceived limitations on the quality of life [e.g., “*Does your health now limit you in these activities (e.g., Lifting or carrying groceries)? If so, how much?”], with a* response scale ranging from *1, Yes, limited a lot*, to *5, No, not limited at all*. The original response scale that ranged from 1 to 3 was adapted for this purpose so that all scales had the same range. The social function (one item) evaluates the amount and level of difficulty to carry out the usual social activities (“*During the past 4 weeks, to what extent has your physical health or emotional problems interfered with your normal social activities with family, friends, neighbors, or groups*?”), with a response scale ranging from *1, not at all*, to *5, extremely*. Physical performance (four items) assesses the limitation on the physical health to perform daily or professional tasks [e.g.„ “*During the past 4 weeks, have you had any of the following problems (e.g., accomplished less than you would like) with your work or other regular daily activities as a result of your physical health*?], with a response scale ranging from *1, yes*, or *2*. General health (five items) rates the overall health consciousness of the individual (e.g., “*In general, would you say your health is*”), with a response scale ranging from *1, excellent*, to *5, fair*. A composite measure of the health-related quality of life was created and revealed an adequate level of internal consistency (α = 0.85).

The summary of participants and procedure is presented in Table 1.

**Table 1:** Summary of participants and procedure.

**Description**	**Sample**	
	**Welder (*N* = 40)**	**Non-welder (*N* = 42)**
**Sociodemographic**		
Male	95%	100%
Age between 30 and 40	40%	100%
Medium level of education	97.5%	83.3%
**Survey measures**		
Musculoskeletal disorders	Yes	Yes
Bodily pain	Yes	No
Health-Related Quality of Life	Yes	No

## Results

### Musculoskeletal Disorders: Comparisons Between Welders and Non-welders

**Figure 1:**
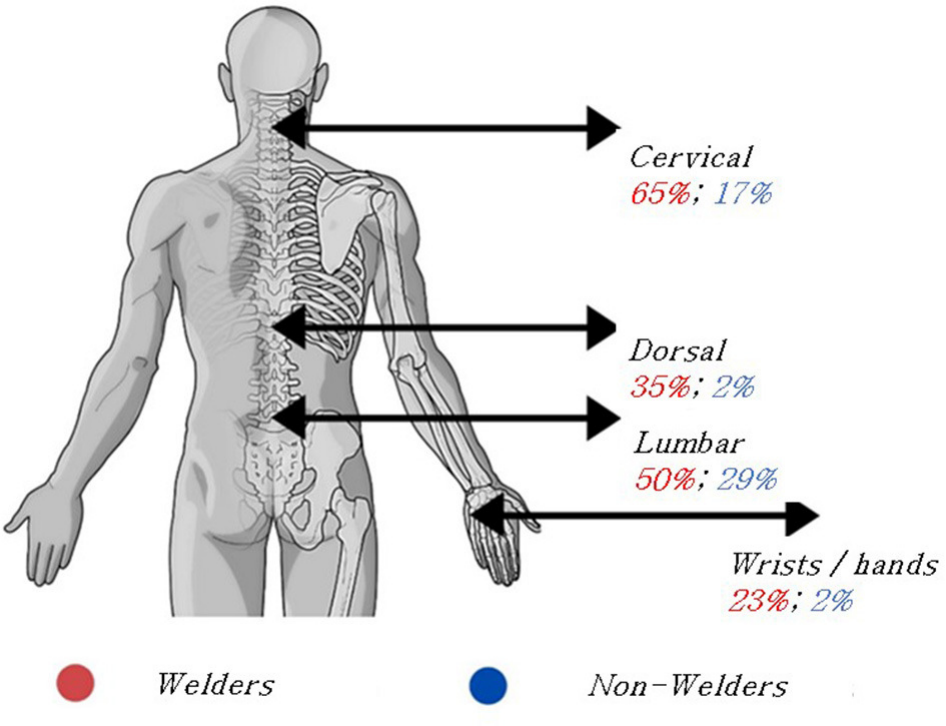
Percentage of incidence of symptoms of musculoskeletal disorders in the areas that were significantly different between welders and non-welders.

The qui-square test was used to compare the frequency of musculoskeletal disorders between welders and non-welders. As predicted, a higher incidence of symptoms of musculoskeletal of disorders was found in welders (Hypothesis 1). In particular, data shows that there is a higher incidence of musculoskeletal disorders in the cervical [χ^2^ = (1, *N* = 82) = 19.90, *p* > 0.001; ϕ = 0.493], lumbar [χ^2^ = (1, *N* = 82) = 3.95, *p* = 0.047, ϕ = 0.220], dorsal [χ^2^ = (1, *N* = 82) = 14.58, *p* < 0.001, ϕ = 0.422], and in the wrists and hands [χ^2^ = (1, *N* = 82) = 7.74, *p* = 0.005, ϕ = 0.307] areas. Among non-welders, there was no incidence of musculoskeletal disorders in any body areas. To control for possible age effects, we further ran the Cochran–Mantel–Haenszel test, distinguishing among the participants below and above 35 years old. The same trend of results emerged. The percentage of welders and non-welders that reported musculoskeletal disorders in those areas is presented in Figure 1.

### Effects of Musculoskeletal Disorders on the Health-Related Quality of Life of Welders, Mediated by Bodily Pain

In average, welders showed a medium level of bodily pain (*Mean* = 3.30, *Standard Deviation* = 1.30) and health-related quality of life (*Mean* = 3.30, *Standard Deviation* = 1.30; both scales ranged between 1 and 5). Most welders reported having two musculoskeletal disorders (35%). To further explore these results, we first calculated the Pearson coefficient to estimate the correlation between quantitative variables and the point-biserial coefficient to estimate the correlation between quantitative and dichotomous variables (i.e., musculoskeletal disorders). The matrix of correlations between variables is presented in Table 2. Only musculoskeletal disorders in the lumbar area were significantly related to higher levels of bodily pain. Bodily pain was very strongly related to a lower health-related quality of life. All musculoskeletal disorders, with the exception of the dorsal area, were related to a significantly lower perception of quality of life.

**Table 2:** Correlation between musculoskeletal disorders, bodily pain, and health-related quality of life.

**Variable**	**1**	**2**	**3**	**4**	**5**	**6**
1. MD cervical	-					
2. MD dorsal	−0.12	-				
3. MD lumbar	0.21	0.42^**^	-			
4. MD wrists/hands	0.14	−0.02	0.18	-		
5. Bodily pain	0.27	0.14	0.47^**^	0.14	-	
6. Quality of life	−0.35^*^	−0.11	−0.43^**^	−0.31^*^	−0.73^***^	-

*MD, musculoskeletal disorders*;

* *p < 0.050*;

** *p < 0.010*;

*** *p < 0.001*.

It was expected that musculoskeletal disorders in welders would cause bodily pain which, in turn, would decrease their health-related quality of life, that is, bodily pain would mediate the relation between musculoskeletal disorders and health-related quality of life in welders (Hypothesis 2). To test the mediation model, we used the bootstrapping method, as it has a relatively higher power for detecting smaller effects. In specific, we used the PROCESS macro for SPSS version 3.5 (15), which is based on the ordinary least square regression and path analysis. The number of bootstrap samples for the percentile bootstrap confidence intervals was 5,000.

The bodily pain was entered as a mediator of the relation between the musculoskeletal disorders in welders (independent variable) and their health-related quality of life (dependent variable). We ran four separate models using as independent variables the musculoskeletal disorders in the cervical, dorsal, lumbar, and in the hands and wrists areas. Hypothesis 2 was partially corroborated. As it was to be expected from the matrix of correlations, we only found a mediation effect of bodily pain on the relation between the incidence of musculoskeletal disorders and health-related quality of life for the lumbar area. In particular, the mediation model explained 54% of the variance of health-related quality of life, *F*(2,37) = 21.59, *p* < 0.001. The indirect effect of bodily pain on the relation between musculoskeletal disorders in the lumbar area and health-related quality of life was significant, −0.55, 95% CI [−0.89, −0.22].

To explore the potential cumulative effects of musculoskeletal disorders in all areas, we computed a new measure that aggregated all musculoskeletal disorders except the lumbar area (cervical, dorsal, and wrists and hands). This new variable varied between 0 (absence of musculoskeletal disorders) and 1 (presence of musculoskeletal disorders in all three areas). A model entering the aggregated measure as an independent variable significantly explained 58% of the variance of health-related quality of life, *F*(2,37) = 25.20, *p* < 0.001. The indirect effect of bodily pain on the relation between the musculoskeletal disorders in all areas, except the lumbar area, and health-related quality of life was significant and slightly higher than the effect obtained in the previous model, −0.69, 95% CI [−1.31, −0.01].

**Figure 2:**
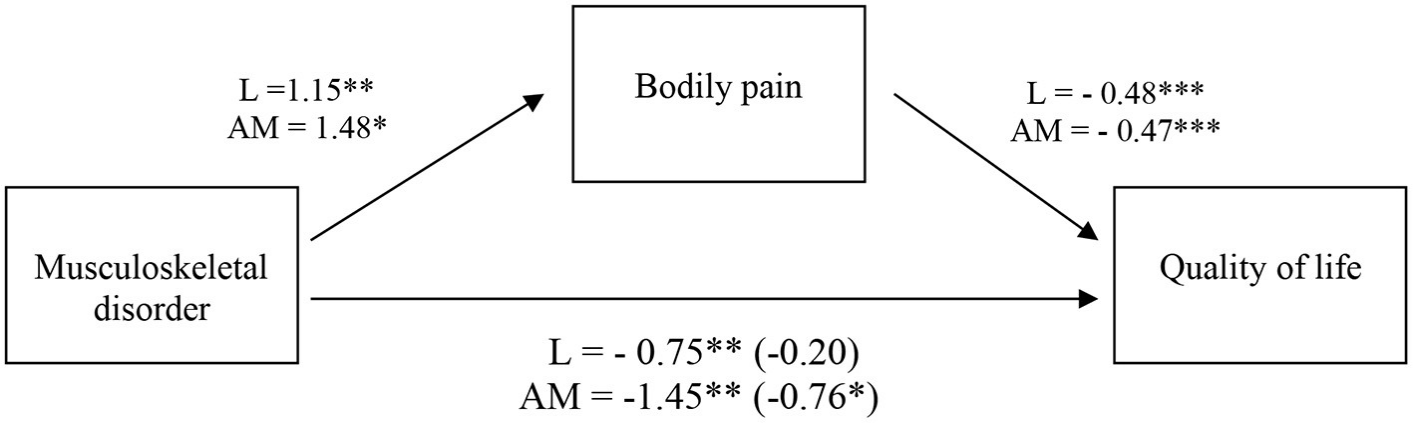
Indirect effect of bodily pain on the relation between musculoskeletal disorders and health-related quality of life. The coefficient for musculoskeletal disorder and health-related quality of life controlling for bodily pain is shown in parenthesis. All values represent unstandardized coefficients. L, lumbar. AM, aggregated measure of musculoskeletal disorders in the cervical, dorsal, and hands and wrists areas. *p < 0.050, **p < 0.010, ***p < 0.001.

This suggests that the musculoskeletal disorder that has more impact on the health-related quality of life due to the effect of bodily pain is located in the lumbar area. However, when considering the cumulative effects of musculoskeletal disorders in the cervical, dorsal, and wrists and hands areas, these were also related to an increase of bodily pain and have a very negative impact on the health-related quality of life. The results of the two mediation models are presented in Figure 2. Of relevance, the relation between musculoskeletal disorders in the cervical, dorsal, and wrists and hands areas and quality of life was only partially mediated by bodily pain. When the mediator is considered in the model, the relation remained significant, though its strength is significantly diminished (from −1.45 to −0.76). This suggests that the other variables that were not considered can further explain the relation between musculoskeletal disorders in those three areas and health-related quality of life.

## Discussion

Welders are exposed to multiple occupational hazards in their workplaces. However, only a few studies have been addressing their health status and quality of life. This study contributes to filling this literature gap by identifying the musculoskeletal disorders in a sample of Portuguese welders and understanding its effects on welders' bodily pain and health-related quality of life. Our findings suggest that welders have a higher incidence of musculoskeletal disorders in the cervical, lumbar, dorsal, and wrists and hands areas than non-welders. These results are only partially convergent with the studies conducted in other countries (2, 3, 8). Although, some of these differences might have to do with the differences in the methods that were used, and differences between arc and gas welding, it is likely that the differences are also linked to the average anthropometric differences between countries. These differences need to be further understood to be considered in the setup of workstations and in the size, weight, and relative dimensions of the welding equipment.

We also found that musculoskeletal disorders in the lumbar area alone, and in the cervical, dorsal, and wrists and hands areas combined, diminished the welders' health-related quality of life. The relation between musculoskeletal disorders in the lumbar area and health-related quality of life was explained by the increased bodily pain. Bodily pain in the lumbar area is a matter of the upmost relevance. The Global Burden of Diseases, Injuries, and Risk Factors Study (16) estimated that low back pain continues to be, since 1990, the fourth leading cause of disability-adjusted life-years (DALYs; i.e., represents the loss of the equivalent of 1 year of full health) for people between 25 and 49 years old (the same age groups of our sample of welders), and has been increasing in other age groups. Pain medication can play an important part in enabling welders to continue working without feeling pain or decreasing their health-related quality of life. However, the widespread use of opioids for the treatment of moderate or severe acute and chronic pain has become a public health problem due to the physical and psychological dependence and tolerance they produce. The increasingly higher doses that patients require may reach toxic levels or lead to accidents, including fatalities (17). Therefore, primary prevention, such as exercise training programs (1, 18), and secondary prevention, as improving the ergonomic design of the welders workstations (19, 20), should be privileged in the future.

## Data Availability Statement

The raw data supporting the conclusions of this article will be made available by the authors, without undue reservation.

## Ethics Statement

Ethical review and approval was not required for the study on human participants in accordance with the local legislation and institutional requirements. The patients/participants provided their written informed consent to participate in this study.

## Author Contributions

LL and SL contributed to conception and design of the study. LL collected the data, performed the statistical analyses and wrote the first draft of the manuscript. SL performed statistical analyses, and wrote sections of the manuscript. All authors contributed to manuscript revision, read, and approved the submitted version.

## Conflict of Interest

The authors declare that the research was conducted in the absence of any commercial or financial relationships that could be construed as a potential conflict of interest.
